# Evolutionary structure constrains genomic prediction accuracy more than model complexity in mango (*Mangifera indica* L.)

**DOI:** 10.1093/g3journal/jkag124

**Published:** 2026-05-11

**Authors:** Ganesan Alagarasan, Abdulqader Jighly, Vanika Garg, Oluwaseun Akinlade, Natalie Dillon, Asjad Ali, Penghao Wang, Christopher I Cazzonelli, Ravi V Mural, Diego Jarquin, Peter Prentis, Rajeev K Varshney

**Affiliations:** WA State Agricultural Biotechnology Centre, Centre for Crop and Food Innovation, Food Futures Institute, Murdoch University, Murdoch, WA 6150, Australia; WA State Agricultural Biotechnology Centre, Centre for Crop and Food Innovation, Food Futures Institute, Murdoch University, Murdoch, WA 6150, Australia; AgriSapiens PTY LTD, Doncaster, VIC 3108, Australia; WA State Agricultural Biotechnology Centre, Centre for Crop and Food Innovation, Food Futures Institute, Murdoch University, Murdoch, WA 6150, Australia; WA State Agricultural Biotechnology Centre, Centre for Crop and Food Innovation, Food Futures Institute, Murdoch University, Murdoch, WA 6150, Australia; Queensland Department of Primary Industries, Mareeba, QLD 4880, Australia; Queensland Department of Primary Industries, Mareeba, QLD 4880, Australia; WA State Agricultural Biotechnology Centre, Centre for Crop and Food Innovation, Food Futures Institute, Murdoch University, Murdoch, WA 6150, Australia; Hawkesbury Institute for the Environment, Western Sydney University, Locked Bag 1797, Penrith, NSW 2751, Australia; Department of Agronomy, Horticulture and Plant Science, South Dakota State University, Brookings, SD 57007, United States; Agronomy Department, University of Florida, Gainesville, FL 32611, United States; Centre for Agriculture and Bioeconomy, Queensland University of Technology, Brisbane, QLD 4001, Australia; WA State Agricultural Biotechnology Centre, Centre for Crop and Food Innovation, Food Futures Institute, Murdoch University, Murdoch, WA 6150, Australia

**Keywords:** genomic prediction, perennial fruit crops, phylogenetic signal, additive genetic architecture, mixed models, kernel methods, ensemble learning, mango traits

## Abstract

In genomic prediction, it remains unclear whether increasingly complex or ensemble models improve prediction over established linear approaches, and why prediction accuracy varies among traits. Here, we evaluated a comprehensive suite of genomic prediction models, including linear mixed models, Bayesian variable selection, kernel methods, machine learning algorithms, graph attention networks, and stacked ensembles, in mango (*Mangifera indica* L.). Across 5 traits, prediction accuracy converged across linear, Bayesian, kernel, and ensemble models, with only marginal gains derived from stacking and no systematic advantage of machine learning approaches. Ensemble ablation and weight analyses revealed that predictive signal was dominated by additive and smooth kernel components, while more complex learners contributed little or negatively upon performance. To explain these trait-dependent patterns in predictability, we quantified the phylogenetic signal using genome-wide marker–based trees. All traits showed a significant phylogenetic signal, with the magnitude varying widely and strongly associated with prediction accuracy (*r* ≈ 0.71). Traits with strong phylogenetic structure achieved the highest prediction accuracies, whereas traits with a weaker signal were consistently harder to predict, regardless of model choice. Together, these results confirm that, in mango, genomic prediction accuracy is determined more by evolutionary structure and trait architecture rather than increasing model complexity. Aligning prediction strategies with the evolutionary basis of trait variation may therefore be more effective than adopting increasingly complex models.

## Introduction

Genomic selection (GS) has transformed modern plant breeding by enabling the prediction of genetic merit using genome-wide molecular markers, thereby reducing phenotyping costs, accelerating breeding cycles, and increasing genetic gain per unit time. Since its formalization more than 2 decades ago, GS has been successfully applied across numerous annual crops and livestock species, consistently outperforming pedigree-based selection for complex traits and traits with limited phenotypic data (Bernardo 1994; Meuwissen et al. 2001; VanRaden 2008; Hickey et al. 2017). Despite these advances, genomic prediction accuracy remains highly context-dependent, varying with trait genetic architecture, population structure, training population design, and the extent of genotype × environment interaction.

Early theoretical and empirical studies established that prediction accuracy depends strongly on assumptions about the distribution of marker effects and trait architecture. Linear mixed models such as genomic best linear unbiased prediction (GBLUP) or its alternative parameterization known as ridge regression BLUP (rrBLUP) perform optimally when traits conform to an infinitesimal architecture dominated by many small-effect loci, offering robustness, low bias, and computational efficiency (Meuwissen et al. 2001; VanRaden 2008; Meher et al. 2022). In contrast, Bayesian shrinkage models such as BayesA, BayesB, and Bayesian LASSO that allow marker-specific variances tend to outperform GBLUP when genetic architectures deviate from strict infinitesimal assumptions, with a subset of markers explaining a disproportionate share of genetic variance, albeit at the cost of increased model complexity and sensitivity to hyperparameter choice (Maltecca et al. 2012; Li et al. 2015; Wang et al. 2018). Hybrid approaches that weight markers within relationship matrices further demonstrate that model performance depends on trait architecture, reinforcing that no single statistical model is universally optimal (Li et al. 2015; Ren et al. 2021). Crucially, to avoid data leakage and inflated accuracy, these importance weights must be derived entirely from the training partition.

Beyond additive genetic assumptions, increasing evidence indicates that nonadditive effects, including dominance and epistasis, may contribute substantially to phenotypic variance in certain traits, particularly in perennial and clonally propagated crops. Nonlinear methods such as reproducing kernel Hilbert space (RKHS) regression, kernel ridge regression, support vector regression (SVR), random forest (RF), and neural networks have been proposed to capture these complex marker interactions (Crossa et al. 2013, 2017; Seyum et al. 2022). While these approaches can improve accuracy for certain traits, their relative advantage over linear models is inconsistent, often diminishing when traits are highly polygenic or when training population sizes are limited. In tropical and perennial crops, genomic prediction suggests differences among individual models are frequently modest, reinforcing the need for strategies that integrate complementary model strengths rather than relying on a single best approach (Seyum et al. 2022; Robinson et al. 2025).

Recent advances have shifted toward ensemble and stacked learning frameworks, which combine predictions from multiple base models to improve robustness and accuracy. Stacking heterogeneous learners, including BLUP-based, Bayesian, and machine-learning models, has repeatedly been shown to outperform individual methods across diverse species and traits, often with reduced overfitting and improved stability across validation scenarios (Liang et al. 2021; Kim et al. 2024). Ensemble methods, such as ELPGV and other stacked generalization frameworks, have demonstrated statistically significant improvements in prediction accuracy in both plant and animal breeding contexts, indicating that model averaging and meta-learning can effectively exploit differences in model inductive bias (Gu et al. 2024; Robinson et al. 2025). Despite this promise, ensemble genomic prediction remains underexplored in perennial fruit crops, particularly those with complex evolutionary histories and diverse geographic origins.

Recent syntheses further identify training population size and genetic diversity as primary determinants of prediction accuracy have diminishing returns beyond an optimum and necessitate an increasing need for optimized model selection in diverse germplasm panels (Alemu et al. 2024). For perennial crops, which are often evaluated across heterogeneous environments and over long-time scales, explicit consideration of G × E and population history is particularly critical (Munyengwa et al. 2025; Robinson et al. 2025; Jighly et al. 2026). However, most genomic prediction studies still treat individuals as independent and interchangeable units, without explicitly examining how evolutionary relatedness or trait divergence influences prediction performance.

Mango (*Mangifera indica* L.) is a globally important perennial fruit crop with high economic, social, and nutritional value, yet its breeding progress is constrained by long generation times, large tree size, and complex trait architectures shaped by domestication, geographic dispersal, and local adaptation (Warschefsky and von Wettberg 2019; Wang et al. 2020). While recent advances in whole-genome sequencing and SNP discovery have enabled genomic prediction in mango, the extent to which different statistical and machine-learning models capture trait-specific genetic signals that remains poorly understood. Moreover, the role of evolutionary relatedness among accessions in shaping genomic prediction accuracy has received little attention in perennial fruit breeding.

In this study, we evaluate a comprehensive suite of genomic prediction models, including linear mixed models, Bayesian shrinkage approaches, nonlinear kernel and machine-learning methods, and stacked ensemble predictors, using a globally diverse, high-density genotyped mango germplasm panel. Specifically, we aim to (i) quantify variation in prediction accuracy across traits with contrasting genetic architectures, (ii) assess whether nonlinear and ensemble models consistently outperform linear baselines, and (iii) explore whether evolutionary relatedness among accessions explains observed differences in prediction performance. By integrating ensemble modeling with phylogenetic analysis, we provide new insights into model choice, trait architecture, and the potential of ensemble genomic prediction to accelerate genetic improvement in the selection of perennial fruit crops.

## Materials and methods

### Plant material and datasets

Genomic prediction analyses were conducted using previously published genotypic and phenotypic data from the Australian Mango Breeding Program (Wilkinson et al. 2024). The dataset comprises 225 *Mangifera indica* accessions originating from 24 countries, maintained at the Walkamin Research Station, Queensland, Australia. Genotypic data were generated via whole-genome resequencing, aligned to the *M. indica* cv. “Alphonso” reference genome, and processed to identify genome-wide SNP markers using a GATK-based variant-calling pipeline (Wilkinson et al. 2024). Phenotypic traits measured in mature trees included fruit blush color (BC), fruit firmness (FF), fruit weight (FW), total soluble solids (TSS), and trunk circumference (TC), averaged across fruits and years where applicable. Full details of population development, sequencing, variant filtering, and phenotyping procedures are described in Wilkinson et al. (2024).

### Genotypic data processing

Raw variant call format (VCF) genotype data were filtered to retain only biallelic single-nucleotide polymorphisms (SNPs) and converted to PLINK binary format using PLINK v2.0 (Chang et al. 2015). Individuals with more than 10% missing genotypes and SNPs with more than 5% missing data were removed. SNPs with a minor allele frequency below 0.05 were excluded, and contigs containing fewer than 50 SNPs were removed to ensure robust imputation. Filtered genotype data were imputed using Beagle v5.4 with default parameters. Imputation output was validated to ensure nonempty genotype calls and consistent marker representation across individuals (Browning et al. 2018). Following imputation, SNP identifiers were standardized to ensure uniqueness. To reduce redundancy among markers, linkage disequilibrium (LD) pruning was performed using PLINK v2.0 with a sliding window of 50 SNPs, and an *r*^2^ threshold of 0.2, resulting in a LD-pruned marker set of 360,138 SNPs for all downstream analyses. For marker matrix construction, imputed and filtered genotypes were encoded as allele dosages and assembled into a marker matrix *M*, with rows representing individuals and columns representing SNPs (Supplementary Table 1).

### Feature selection using random forests

To reduce marker dimensionality prior to genomic prediction, SNP feature selection was performed separately for each trait using an RF regression approach implemented in Python. RF was selected for feature selection due to its ability to capture both linear and nonlinear marker–trait relationships and to provide stable, model-agnostic importance rankings without strong parametric assumptions. Individuals were matched between genotype and phenotype datasets using unique identifiers, and analyses were conducted using only individuals with nonmissing phenotypes for the focal trait. Because the genotype matrix contained several hundred thousand SNP columns, RF model fitting was conducted using a 2-stage, chunked procedure to manage computational complexity. In the first stage, SNP columns were processed in sequential blocks of 30,000 markers. Within each block, an RF model (500 trees; max_features = sqrt; min_samples_leaf = 2) was trained to predict the trait value. SNP importance was quantified using the scikit-learn Gini-based feature importance (mean decrease in impurity), and the top 1,000 SNPs per block were retained as candidate markers and pooled across blocks with duplicates removed. In the second stage, all candidate SNPs identified in stage 1 were jointly refit in a final RF model (600 trees; max_features = sqrt; min_samples_leaf = 2) and ranked by feature importance. For each trait, the top 10,000 SNPs were retained for downstream genomic prediction analyses. This RF-based SNP selection was conducted once per trait as a global dimensionality-reduction step before genomic prediction and was not repeated across cross-validation folds. We acknowledge that performing feature selection on the full dataset prior to cross-validation may introduce minor information leakage and slightly inflate absolute prediction accuracy. This was adopted as a pragmatic compromise for computational feasibility. Nevertheless, all models were evaluated using a fully nested cross-validation framework, ensuring unbiased comparison of predictive performance.

### Genomic prediction models

**Fig. 1.**
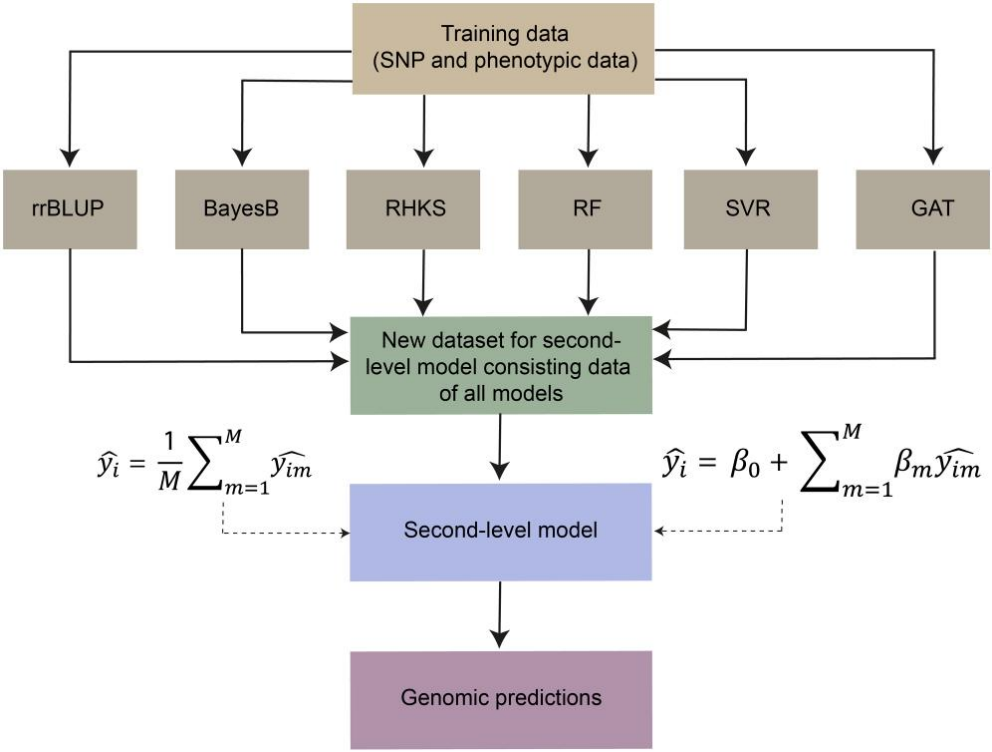
Workflow of the ensemble genomic prediction framework. SNP and phenotypic data are used to train multiple base models (rrBLUP, BayesB, RKHS, RF, SVR, and GAT). Predictions from these models are combined to construct a second-level dataset, which is then used in a meta-model to generate final genomic predictions.

Genomic prediction was conducted using a 2-level ensemble modeling framework. In the first level, multiple base genomic prediction models (base learners) were independently trained using SNP and phenotypic data. These base models included ridge regression best linear unbiased prediction (rrBLUP), Bayesian regression (BayesB), RKHS regression, RF, SVR, and graph attention network (GAT) (Henderson 1975; Cortes and Vapnik 1995; Breiman 2001; Meuwissen et al. 2001; Gianola and Van Kaam 2008). Each base model produced genomic predictions for the target trait. Predictions from all first-level models formed inputs to second-level meta-models. Two different meta-models were evaluated at this second level to integrate predictions across base models and generate final genomic predictions (Fig. 1).

Let *n* denote the number of individuals and *p* the number of markers and


$$y=(y_{1},\ldots ,y_{n}{)}^{⊤}$$

denote the vector of phenotypic observations, *μ* the overall mean, and Z the n×p matrix of genotype covariates encoded as additive allele dosages. Predicted phenotypic values (genomic predictions) for individual *i* are denoted by ${\hat{y}}_{i}$.

Genomic prediction was performed using multiple statistical and machine-learning models. All models were trained using the same training data and produced predictions on a common scale, enabling direct comparison and subsequent ensemble learning.

#### Ridge regression best linear unbiased prediction

rrBLUP models the relationship between phenotypes and genome-wide markers using a linear mixed model (Henderson 1975):


y=1μ+Zu+ε

where u is the vector of marker effects and ε is the vector of residual errors. Jointly, marker effects are assumed to follow a normal distribution


$$u∼N(0,\sigma_{u}^{2}I)$$

and residuals are assumed to be independent and normally distributed


$$\varepsilon ∼N(0,\sigma_{e}^{2}I)$$

Under these assumptions, rrBLUP applies uniform shrinkage to all marker effects. Genomic prediction values are obtained as:


$$\hat{y}=Z\hat{u}$$

#### Bayesian regression model

BayesB is a Bayesian variable selection regression model that assumes only a subset of markers have nonzero effects. The model is defined as (Meuwissen et al. 2001)


y=1μ+Zu+ε

Each marker effect $u_{j}$ follows a mixture prior


$$u_{j}∼\{\begin{array}{cc} 0, & \mathrm{with}\;\mathrm{probability}\;\pi \\ N(0,\sigma_{u_{j}}^{2}), & \mathrm{with}\;\mathrm{probability}\;1-\pi \end{array}$$

where *π* represents the prior proportion of markers assumed to have zero effect. Here, *π* was fixed at 0.95, implying that 95% of SNPs were assumed a priori to have no effect on the trait.

Posterior distributions of marker effects are estimated using Markov chain Monte Carlo sampling. Genomic prediction values are obtained as


$$\hat{y}=Z\hat{u}$$

#### RKHS regression

RKHS regression is a semi-parametric approach that captures nonlinear relationships between genotypes and phenotypes using kernel functions (Gianola and Van Kaam 2008). The model is expressed as


y=1μ+g+ε

where g represents the vector of genetic effects and is assumed to follow


$$g∼N(0,K\sigma_{g}^{2})$$

Here, K is a kernel matrix derived from genome-wide marker data. A Gaussian kernel was used


$$K_{ij}=\exp\left(-\frac{d_{ij}^{2}}{2\theta^{2}}\right)$$

where $d_{ij}$ denotes the Euclidean distance between individuals *i* and *j* based on marker genotypes, and *θ* is a bandwidth parameter.

Genomic predictions are given by


$$\hat{y}=\hat{g}+1\hat{\mu }$$

#### RF regression

RF regression is a nonparametric ensemble method that aggregates predictions from multiple decision trees (Breiman 2001). For individual *i*, the RF prediction is given by


$${\hat{y}}_{i}=\frac{1}{T}\overset{T}{\underset{t=1}{\sum }}\;f_{t}(z_{i})$$

where *T* is the number of trees in the forest, $z_{i}$ is the genotype vector for individual *i*, and $f_{t}(\cdot )$ denotes the prediction from the *t*th regression tree. Each tree is trained on a bootstrap sample of the data, and random subsets of markers are considered at each split. Final predictions are obtained by averaging predictions across all trees.

#### Support vector regression

SVR is a kernel-based method that estimates a function of the form (Cortes and Vapnik 1995)


$$f(z)=w^{⊤}\phi (z)+b$$

where ϕ(⋅) maps genotypes into a high-dimensional feature space. Model parameters are estimated by minimizing the regularized ε -insensitive loss function:


$$\underset{w,b}{\min}\;\frac{1}{2}∥w{∥}^{2}+C\overset{n}{\underset{i=1}{\sum }}\;(\xi_{i}+\xi_{i}^{*})$$

subject to standard SVR constraints. Using the kernel trick, predictions are expressed as:


$${\hat{y}}_{i}=\overset{n}{\underset{\;j=1}{\sum }}\;\alpha_{j}K(z_{i},z_{j})+b$$

where K(⋅,⋅) is a kernel function and $\alpha_{j}$ are learned coefficients.

#### Graph attention network

In GAT, each individual is represented as a node with a marker-derived feature vector $z_{i}$, and edges are constructed using a *k*-nearest neighbor graph based on Euclidean distance in genotype space.

Let


$$h_{i}^{\left(0\right)}=z_{i}$$

denote the initial node features.

At layer *l*, node representations are updated using attention-weighted aggregation of neighboring nodes


$$h_{i}^{\left(l+1\right)}=\sigma \left(\underset{\;j\in N(i)}{\sum }\;\alpha_{ij}^{\left(l\right)}W^{\left(l\right)}h_{j}^{\left(l\right)}\right)$$

where $W^{\left(l\right)}$ is a learnable weight matrix, N(i) denotes the set of neighbors of node *i*, σ(⋅) is a nonlinear activation function, and $\alpha_{ij}^{\left(l\right)}$ represents the attention coefficient between nodes *i* and *j*.

Attention coefficients are computed as


$$\alpha_{ij}^{\left(l\right)}=\frac{\exp(\mathrm{LeakyReLU}(a^{(l)⊤}[W^{\left(l\right)}h_{i}^{\left(l\right)}∥W^{\left(l\right)}h_{j}^{\left(l\right)}]))}{\sum_{k\in N(i)}\;\exp(\mathrm{LeakyReLU}(a^{(l)⊤}[W^{\left(l\right)}h_{i}^{\left(l\right)}∥W^{\left(l\right)}h_{k}^{\left(l\right)}]))}$$

where $a^{\left(l\right)}$ is a learnable attention vector and ∥ denotes concatenation.

A multihead attention mechanism is used in the first layer


$$h_{i}^{\left(1\right)}={∥}_{m=1}^{M}\sigma \left(\underset{\;j\in N(i)}{\sum }\;\alpha_{ij}^{\left(1,m\right)}W^{\left(1,m\right)}h_{j}^{\left(0\right)}\right)$$

A second attention layer aggregates the representations into a latent embedding $h_{i}^{\left(2\right)}$, which is passed through a linear output layer to obtain genomic prediction values


$${\hat{y}}_{i}=w^{⊤}h_{i}^{\left(2\right)}+b$$

#### Stacked ensemble meta-models

To integrate predictions from all base models, a stacked ensemble framework was implemented (Wolpert 1992; Nascimento et al. 2024). Let ${\hat{y}}_{im}$ denote the prediction for individual *i* obtained from the *m*th base model, where m=1,…,M.

Linear stacking meta-model


$${\hat{y}}_{i}=\beta_{0}+\overset{M}{\underset{m=1}{\sum }}\;\beta_{m}{\hat{y}}_{im}$$

where $\beta_{0}$ is the intercept and $\beta_{m}$ represents the weight assigned to the *m*th base model.

Mean ensemble meta-model


$${\hat{y}}_{i}=\frac{1}{M}\overset{M}{\underset{m=1}{\sum }}\;{\hat{y}}_{im}$$

This unweighted ensemble assigns equal importance to all base models and serves as a baseline for comparison with the linear stacking approach.

### Cross-validation framework and evaluation strategy

All models were evaluated using a fully nested cross-validation framework to obtain unbiased estimates of predictive performance and to prevent information leakage at every stage of model training and ensemble construction. All hyperparameters were fixed a priori, and no additional tuning was performed during cross-validation. Analyses were conducted separately for each trait and were restricted to individuals with nonmissing phenotypic records. Model evaluation was based on repeated outer cross-validation combined with an inner cross-validation used for ensemble training. In the outer loop, the dataset was randomly partitioned into 5 approximately equal folds. In each outer iteration, 1-fold was held out as an independent test set, while the remaining 4 folds were used for model training. This 5-fold outer cross-validation procedure was repeated 10 times using independent random fold assignments to improve robustness of performance estimates. Within each outer training set, an inner 5-fold cross-validation was performed to generate out-of-fold predictions for each base model. Specifically, the outer training data were further divided into inner folds, and each base learner was trained on 4 inner folds and used to predict the held-out inner fold. This process was repeated until all individuals in the outer training set received predictions from models that were not trained on their own phenotypic values. The resulting out-of-fold predictions from all base models constituted the training data for the ensemble meta-models used in stacking. Only these out-of-fold predictions were used to fit the meta-models, ensuring that no individual contributed both training information and prediction targets to the same model and thereby preventing optimistic bias. After fitting the meta-models, all base learners were retrained on the full outer training set and used to generate predictions for the outer test set. These test-set base-model predictions were then combined using the previously trained meta-models to produce final ensemble predictions for the held-out individuals.

### Performance assessment, bias diagnostics, and ablation analyses

Model performance was evaluated exclusively on the outer test folds. The primary performance metric was the Pearson correlation between observed and predicted phenotypes. To assess whether differences in predictive performance between models were statistically significant, paired Wilcoxon signed-rank tests were performed across cross-validation replicates. Additional error-based metrics, including root mean squared error and mean absolute error, were also computed. Spearman rank correlation was calculated as a supplementary measure of predictive concordance. Performance metrics were first computed separately for each outer fold and repetition. Overall performance estimates were obtained by averaging metrics across all outer folds and repetitions for each model and trait. To illustrate the importance of proper nesting in stacked ensembles, an additional non-nested stacking procedure was evaluated as a diagnostic comparator. In this analysis, the meta-model was trained using in-sample base-model predictions derived from the outer training data, rather than out-of-fold predictions. This approach was used solely to demonstrate the optimistic bias that arises when stacking is performed without appropriate cross-validation. In addition, leave-one-model-out ablation analyses were conducted for the stacked ensemble. Each base learner was removed in turn from the stacking framework, and the meta-model was refit using the remaining base models. Changes in predictive performance relative to the full ensemble were used to assess the marginal contribution of each base model.

### Phylogenetic signal

Genetic distances among lines were computed from genome-wide markers (LD-pruned) to construct a neighbor-joining tree to approximate the genetic relationships. Phenotypic values for each trait were matched to the tree tips. We quantified phylogenetic signal using Blomberg's *K* and Pagel's *λ*. Blomberg's *K* compares the variance of phylogenetically independent contrasts to what would be expected if the trait evolved by Brownian motion. Under the Brownian motion model, *K* = 1. If *K* < 1, the trait exhibits less phylogenetic signal than expected under Brownian motion (close relatives are less similar than predicted), whereas *K* > 1 indicates more phylogenetic signal than expected (close relatives are unusually similar) (Münkemüller et al. 2012). This statistic can be interpreted as the partitioning of variance: when *K* > 1, variance tends to be distributed among clades rather than within clades. We estimated *K* using 1,000 random tip-swap permutations to obtain a *P*-value for the null hypothesis of no phylogenetic signal. Pagel's *λ* is a branch-length transformation applied to the tree; internal branches are multiplied by *λ* such that *λ* = 1 yields the original tree (Brownian motion) and *λ* = 0 collapses the tree to a star phylogeny, implying trait values are independent of phylogeny. Thus, *λ* naturally ranges from 0 (no phylogenetic signal) to 1 (strong signal consistent with Brownian motion), with intermediate values indicating partial signal. We estimated *λ* by maximum likelihood and compared it to a model with *λ* = 0 using a likelihood-ratio test; a small *P*-value indicates that *λ* differs significantly from zero.

Computational scripts were developed with assistance from OpenAI Codex to improve coding efficiency. Text refinement and grammar correction were performed using Grammarly. In all cases, outputs were independently assessed, corrected where necessary, and validated by the authors prior to use in analysis and reporting.

## Results

### Genomic prediction across models and traits

Among the 6 base genomic prediction models evaluated for 5 traits: 3 linear/kernel methods (rrBLUP, BayesB, RKHS), 2 machine-learning methods (RF, SVR), and a graph attention network (GAT). In addition, 2 ensemble approaches were assessed as meta-model predictors: a simple mean ensemble (MeanEnsemble) and a ridge-regression stacking model (Stacking_Ridge).

**Fig. 2.**
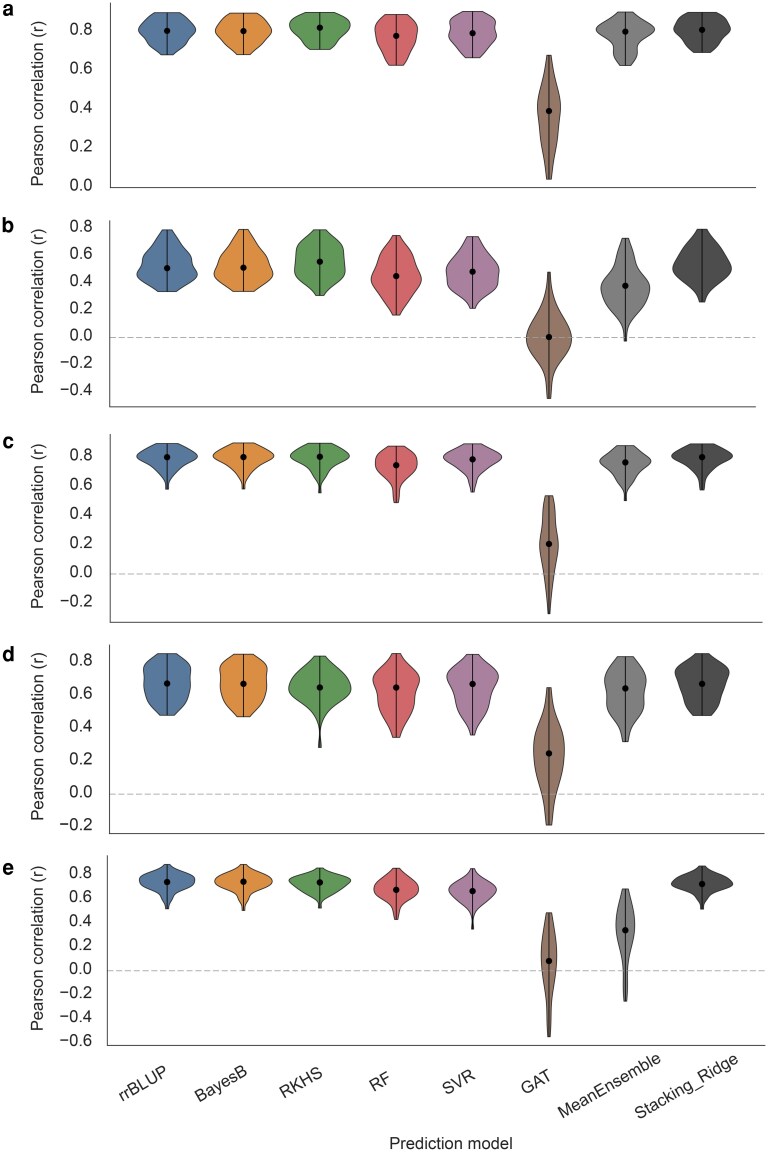
Distribution of genomic prediction accuracy across models. Violin plots show Pearson's correlation coefficient (*r*) between observed and predicted phenotypic values for different prediction models across multiple traits. The width of each violin represents the density of prediction accuracy, with central points indicating the median performance. Here, a is blush color, b is fruit firmness, c is fruit weight, d is trunk circumference, and e is total soluble solids.

Across traits, linear and kernel-based models consistently achieved the highest predictive accuracy for fruit BC, RKHS achieved the highest Pearson (*r* = 0.812) and Spearman (*ρ* = 0.792) correlations, indicating strong agreement between predictions and observations. Stacking_Ridge (*r* ≈ 0.806) and rrBLUP (*r* ≈ 0.800) performed comparably, whereas SVR and RF showed moderately lower correlations (*r* ≈ 0.77 to 0.79). The GAT model performed poorly (*r* ≈ 0.36), with substantially larger root mean square error (RMSE) and mean absolute error (MAE). For the top-performing models, RMSE and MAE were approximately 8.2 and 6.2, respectively (Fig. 2a).

Prediction accuracy for FF was lower overall. RKHS again yielded the highest Pearson (*r* = 0.558) and Spearman (*ρ* = 0.550) correlations, followed closely by Stacking_Ridge (*r* ≈ 0.540) and rrBLUP (*r* ≈ 0.532). RF and SVR achieved lower correlations (*r* ≈ 0.46 to 0.48). GAT produced near-zero correlations and large prediction errors, indicating a failure to capture trait variation. RMSE values for the best models ranged from *r* ∼ 0.217 to 0.222 (Fig. 2b). For FW, BayesB achieved the highest Pearson correlation (*r* = 0.795), with RKHS and rrBLUP performing similarly (*r* ≈ 0.794 and 0.792). SVR and RF showed slightly lower correlations (*r* ≈ 0.74 to 0.78). Ensemble models yielded intermediate performance, while GAT again performed poorly (*r* ≈ 0.21). RMSE values for the top models (∼2.39) were lower than those of the machine-learning approaches (Fig. 2c).

For TC, rrBLUP provided the highest predictive ability (*r* ≈ 0.675). BayesB (*r* ≈ 0.672), Stacking_Ridge (*r* ≈ 0.668), and RKHS (*r* ∼ 0.649). RF and SVR achieved correlations of approximately 0.62 to 0.64, whereas GAT again performed poorly (≈0.23). RMSE and MAE values for the best models were ∼7.74 and ∼6.16, respectively, with only minor differences among the top linear methods (Fig. 2d). For TSS, rrBLUP produced the highest Pearson (*r* = 0.738) and Spearman (*ρ* = 0.743) correlations. BayesB and Stacking_Ridge showed comparable performance (*r* ≈ 0.737 and 0.729), while RKHS and RF achieved moderate correlations (≈0.725 and 0.678). SVR and GAT performed poorly (≈0.659 and 0.068). The best models achieved very low RMSE (∼0.060) and MAE (∼0.047) (Fig. 2e).

### Comparative performance across models

To summarize overall performance, we calculated the average Pearson correlation across the 5 traits for each model (Table 1). The linear and kernel-based models (rrBLUP, BayesB, and RKHS) as well as the ridge-based stacking ensemble consistently achieved the highest predictive ability, with mean correlations around 0.70. Differences among these methods were small, suggesting limited gains from model complexity for the traits studied. Ensemble methods did not yield substantial improvements over the best individual models. This reflects the similarity in predictive signal captured by the base models, limiting the potential for stacking to provide additional gains. RF and SVR achieved moderate predictive ability (0.65 to 0.67) but did not match the performance of linear or kernel-based methods. MeanEnsemble performed the worse (0.57), and the GAT showed very low correlations and substantially higher RMSE and MAE values across all traits (0.18). The traits such as BC, FW and TSS exhibited higher predictive accuracy (≈0.74 to 0.81), whereas FF showed the lowest accuracy (≈0.56). Paired comparisons across CV replicates revealed that several pairwise differences between models were statistically significant (*P* < 0.001), particularly for RF, SVR, and GAT, which consistently underperformed relative to rrBLUP (Supplementary Table 2). However, differences among linear, Bayesian, kernel-based, and stacking models were small and inconsistent across traits, indicating no systematic advantage of more complex approaches.

**Table 1.** Average Pearson correlation across traits.

Model	Mean r	Summary of performance
rrBLUP	0.708	Best for TC and TSS; competitive for FW and BC
BayesB	0.707	Best for FW; similar to rrBLUP and RKHS
RKHS	0.708	Best for BC and FF; tied for FW
Stacking_Ridge	0.707	Ensemble approach; matches linear methods
SVR	0.671	Slightly lower performance; sensitive to trait scale
RF	0.652	Lower correlations, particularly on BC and TSS
MeanEnsemble	0.570	Averaging predictions diluted performance
GAT	0.175	Poor performance across all traits

### Ablation experiments and stacking analysis

#### Stacking framework comparison

To assess the sensitivity of the choice of stacking framework, we compared multiple meta-model frameworks: nested elastic-net, ordinary least squares, and ridge regression meta-models (with and without scaling), as well as a non-nested ridge approach. All frameworks produced similar mean Pearson correlations across traits (≈0.701 to 0.707). The nested ridge model with scaling achieved the highest mean correlation (∼0.7066), although differences among frameworks were negligible (Table 2).

**Table 2.** Mean Pearson correlation across traits for different stacking frameworks.

Framework	Mean *r*
NESTED_RIDGE_SCALE	0.7066
NESTED_RIDGE_NOSCALE	0.7065
NESTED_ENET05_SCALE	0.7055
NESTED_OLS_SCALE	0.7018
NONNESTED_RIDGE_OPTIMISTIC	0.7009

#### Leave-one-model-out ablation

To quantify the contribution of individual base models to the stacking ensemble, we performed leave-one-model-out ablation. Removing RKHS resulted in the largest reduction in predictive ability (Δ*r* = +0.0076), indicating that RKHS was the most informative contributor (Table 3). Removing rrBLUP or BayesB caused only minor decreases in performance, whereas removing RF, SVR or GAT slightly improved correlation, suggesting that these models introduced noise into the ensemble. These results show that RKHS contributes the most to ensemble performance, followed by rrBLUP and BayesB. Conversely, the GAT, random forest, and SVR often reduce predictive ability, particularly for certain traits.

**Table 3.** Average change in Pearson correlation (Δ*r*) when a base model is removed.

Removed model	Δ*r*	Interpretation
NO_RKHS	+0.0076	RKHS is the most beneficial contributor
NO_rrBLUP	+0.0009	Modest positive contribution
NO_BayesB	+0.0007	Modest positive contribution
NO_RF	−0.0006	RF slightly degrades performance
NO_SVR	−0.0017	SVR can be detrimental
NO_GAT	−0.0033	GAT predictions are generally harmful

Note: Positive Δ*r* indicates that the removed model contributed positively to correlation.

### Stacking weights

Analysis of stacking weights showed that the meta-model consistently emphasized linear methods (rrBLUP, BayesB, and RKHS), which received positive weights in ≥99% of cross-validation replicates (Table 4). SVR received positive weights in ∼76% of replicates, while RF and GAT were positive in ∼49% and ∼62%, respectively. Across traits, stacking weights were largest for linear methods, whereas RF and GAT often received near-zero or negative weights, confirming their limited contribution to ensemble performance.

**Table 4.** Mean (SD) stacking weights for each base model across traits.

Model	BC	FF	FW	TC	TSS
BayesB	2.149 (0.508)	0.032 (0.018)	0.908 (0.160)	2.062 (0.615)	0.024 (0.005)
GAT	0.401 (0.602)	−0.002 (0.018)	0.008 (0.217)	0.090 (0.610)	0.001 (0.005)
RF	0.496 (1.037)	−0.018 (0.031)	0.048 (0.307)	−0.044 (1.169)	−0.003 (0.009)
RKHS	3.699 (0.801)	0.096 (0.035)	0.899 (0.193)	1.485 (0.878)	0.016 (0.008)
SVR	2.570 (0.873)	−0.010 (0.040)	0.361 (0.264)	0.943 (0.693)	−0.001 (0.006)
rrBLUP	2.023 (0.502)	0.032 (0.018)	0.834 (0.188)	2.182 (0.683)	0.025 (0.006)

### Phylogenetic signal analysis and implications for predictive accuracy

All traits exhibited statistically significant phylogenetic signal (Table 5), with permutation tests for Blomberg's *K* yielding *P* = 0.001 and likelihood-ratio tests for Pagel's *λ* indicating that *λ* differed significantly from zero (*P* ranging from 10^−4^ to 10^−32^). Because phylogenetic trees were constructed using trait-specific SNP subsets, these estimates should be interpreted within traits rather than as directly comparable across traits. BC and FW showed strong phylogenetic signal (*K* ≈ 1.27; *λ* ≈ 0.87 to 0.99), indicating that closely related genotypes were more similar than expected under Brownian motion. TSS and TC exhibited intermediate signal, whereas FF showed the weakest signal (*K* ≈ 0.67; *λ* ≈ 0.56). Within each trait, stronger phylogenetic signal was associated with higher predictive accuracy; for example, BC and FW achieved high predictive accuracy (r ≈ 0.80), suggesting that additive genetic effects captured by genome-wide markers explain a large proportion of phenotypic variation. TC and TSS showed intermediate signal and intermediate predictive ability, and FF exhibited weaker phylogenetic signal and the lowest predictive accuracy (*r* ≈ 0.56), consistent with greater within-clade variation and a larger contribution of nonadditive genetic effects or environmental influences. Overall, these results indicate that genomic prediction accuracy is closely linked to the extent to which trait variation aligns with genetic relationships captured by trait-relevant markers. Traits with high *K* and *λ* values are more amenable to accurate prediction using genome-wide markers, whereas traits with weaker phylogenetic structure tend to be more labile and difficult to predict. Phylogenetic trees revealed a clear hierarchical structure among genotypes, with multiple well-supported clades and substantial within-clade diversity (Supplementary Figs. 1 to 5). These patterns indicate that variation in Blomberg's *K* and Pagel's *λ* reflects differences in how individual traits align with underlying genomic relatedness within each trait-specific marker set.

**Table 5.** Phylogenetic signal statistics for each trait.

Trait	Blomberg's *K* (*P*)	Pagel's *λ* (*P*)
BC	1.267 (0.001)	0.866 (5.48 × 10^−32^)
FF	0.669 (0.001)	0.556 (7.07 × 10^−5^)
FW	1.268 (0.001)	0.987 (1.65 × 10^−28^)
TC	0.829 (0.001)	0.576 (5.27 × 10^−12^)
TSS	0.896 (0.001)	0.750 (7.81 × 10^−19^)

## Discussion

### Genomic prediction accuracy is primarily shaped by trait architecture and population structure

We evaluated a diverse set of genomic prediction models and ensemble stacking approaches across multiple traits in mango. Despite methodological diversity among these models, prediction accuracy converged across most approaches, with rrBLUP, BayesB, RKHS, and ridge-based stacked ensembles achieving nearly identical levels of accuracy for most traits. This convergence is not unexpected and is consistent with a growing body of theoretical and empirical evidence indicating that, for moderately polygenic traits evaluated within related populations, model choice explains considerably less variation in predictive performance than factors such as trait genetic architecture, training population composition, and realized genomic relatedness.

Foundational studies have demonstrated that when traits are controlled by a large number of loci with small individual effects, genomic prediction methods tend to show similar performance because they ultimately rely on comparable sources of information captured through genome-wide relatedness matrices (Daetwyler et al. 2013; Calus et al. 2014; Gianola et al. 2014). Under such conditions, differences among linear, Bayesian, and kernel-based regression models are often obscured by cross-validation variability, with prediction accuracy approaching an upper limit defined by trait heritability and the degree of correspondence between training and validation populations. The results of the present study are in agreement with these expectations and further support the view that, in many breeding scenarios, rrBLUP and closely related methods already extract most of the usable additive genetic signal from dense SNP datasets.

Importantly, variation in prediction accuracy was observed to be more pronounced across traits than across most models. Traits such as FW and blush color exhibited substantially higher predictability than FF, a pattern that closely reflects their contrasting levels of phylogenetic signal. Traits with stronger phylogenetic signal (high *K* and *λ*) are likely to exhibit phenotypic variation that aligns more closely with genome-wide relatedness, thereby enabling additive models to capture a larger proportion of the underlying genetic variance. In contrast, traits characterized by weaker phylogenetic signal may be more strongly influenced by micro-environmental effects, measurement error, physiological thresholds, or nonadditive genetic interactions, all of which tend to reduce prediction accuracy irrespective of model complexity. Similar trait-dependent differences in genomic prediction performance have been widely reported across crop and livestock species and are recognized as a major determinant of prediction success (Heslot et al. 2012; Rincent et al. 2012; Yannam et al. 2025).

### Limited differentiation among linear, Bayesian, and kernel models reflects shared inductive structure

Although Bayesian variable selection models are generally expected to outperform rrBLUP when large-effect loci exist, the results of the present study revealed only a marginal advantage of BayesB over rrBLUP or RKHS. This finding suggests that the evaluated traits are predominantly polygenic in nature, with few, if any, loci exerting major effects that are detectable given the available sample size. Under such genetic architectures, shrinkage-based and variable-selection approaches tend to converge toward similar predictive performance, a pattern that has been well documented in both simulation-based and empirical investigations (Daetwyler et al. 2013; Calus et al. 2014).

Kernel-based methods such as RKHS are, in principle, capable of capturing nonlinear relationships and local epistatic interactions among loci. However, empirical improvements associated with kernelization are often limited unless strong nonadditive genetic effects are present and are adequately represented within the training population. In this study, RKHS consistently exhibited competitive prediction accuracy but did not show systematic superiority over linear or Bayesian models, indicating that any underlying nonlinear effects were either weak, strongly correlated with additive genetic structure, or insufficiently sampled. This interpretation is consistent with previous reports suggesting that kernel methods tend to enhance model robustness rather than substantially increase absolute prediction accuracy, and that their relative advantage diminishes as training population size decreases or as trait architecture becomes largely additive (Gianola et al. 2014; Azodi et al. 2019).

### Effect of SNP feature selection on model convergence

The convergence in predictive performance observed among linear, Bayesian, kernel-based, and ensemble models in the present study is likely to have been influenced by the SNP feature selection strategy adopted prior to model fitting. Feature selection substantially reduces the dimensionality of the genotype matrix and enriches the retained marker set for loci exhibiting relatively strong marginal effects. As a result, the signal-to-noise ratio is improved, and the differences among alternative modeling frameworks are correspondingly reduced. Under such circumstances, shrinkage-based linear models, Bayesian variable selection approaches, and kernel regression methods are expected to converge in prediction accuracy, as they primarily exploit overlapping additive genetic signal captured within the curated subset of SNP markers.

Empirical benchmarking studies have consistently demonstrated that SNP subsetting diminishes the relative advantage of more complex machine-learning and ensemble-based approaches, particularly in small- to moderate-sized training populations, where high-capacity models would otherwise depend on implicit feature discovery to outperform simpler regression-based methods (Azodi et al. 2019; Banerjee et al. 2020; Mora et al. 2023; Al-Mamun et al. 2025). In addition, feature selection can reduce prediction diversity among individual base learners by aligning their inductive biases, thereby restricting the potential for ensemble stacking to generate further improvements in predictive performance (Heilmann et al. 2023; Tomura et al. 2025). Consequently, the similarity in model performance observed in this study is more likely to reflect a biologically and statistically informed analytical design rather than a lack of methodological distinction among the evaluated models. This outcome is consistent with expectations for genomic prediction pipelines that prioritize robustness, interpretability, and computational tractability, particularly in applied breeding contexts.

### Ensemble stacking provides diagnostic insight but limited accuracy gains

Ensemble learning approaches have been widely proposed as a means of integrating complementary strengths of individual models in order to enhance predictive performance. In our study, ensemble stacking achieved prediction accuracy comparable to that of the best-performing individual models; however, it did not result in consistent improvements across traits. Analyses based on ensemble ablation and stacking weights indicated that the majority of the predictive signal was contributed by rrBLUP, BayesB, and RKHS, whereas machine-learning and graph-based models provided minimal contribution and, in certain cases, exerted a negative influence on prediction accuracy.

This outcome is in close agreement with findings from ensemble learning theory and empirical studies, which indicate that the success of ensemble approaches depends critically on the diversity and relative independence of the constituent base learners (Tomura et al. 2025). When component models are characterized by highly correlated inductive biases, as is typically the case for linear, Bayesian, and kernel-based genomic prediction methods, the potential for stacking to deliver additional gains is inherently limited. Empirical investigations across plant and animal breeding systems further support this view, demonstrating that ensemble methods tend to outperform single-model approaches primarily in contexts involving strong nonadditive genetic effects, complex hybrid population structures, or highly unbalanced datasets (Heilmann et al. 2023; Mora et al. 2023). Rather than interpreting the modest gains from stacking as a limitation, the results of the present study underscore the utility of ensemble methods as valuable diagnostic tools. The estimated stacking weights and ablation effects provide interpretable insights into the relative importance of different modeling assumptions for a given trait–population combination. In this study, such analyses consistently highlighted the predominance of additive and smooth kernel-based genetic signal, thereby reinforcing conclusions drawn from the performance of individual models. Moreover, across all evaluated scenarios, ridge-based stacking achieved prediction accuracy comparable to that of the top-performing individual model for all traits.

### Machine learning and deep learning models are constrained by sample size and data representation

In our study, machine-learning and graph neural network models exhibited inferior predictive performance when compared with simpler genomic prediction methods. This observation should not be interpreted as evidence against the utility of advanced modeling approaches in general, but rather as a reflection of well-recognized constraints associated with genomic prediction. High-capacity models typically require large training populations in order to avoid overfitting, and their performance is highly sensitive to factors such as feature selection, hyperparameter optimization, and data representation (Azodi et al. 2019; Banerjee et al. 2020).

Recent comparative investigations have consistently reported that deep learning models frequently fail to outperform GBLUP for additive or moderately complex traits and may, in some cases, result in lower predictive correlations, even when loss-based performance metrics are comparable or improved (Nazzicari and Biscarini 2022). This apparent discrepancy arises because deep learning models often optimize objective functions that are not well aligned with correlation-based accuracy measures commonly employed in GS. Furthermore, in the absence of carefully engineered data representations, such as biologically informed kinship matrices or haplotype-based features, deep models face substantial difficulty in extracting meaningful genetic signal from high-dimensional SNP datasets, particularly at modest sample sizes. The findings of our study therefore highlight an emerging consensus that increased model complexity, in itself, does not necessarily translate into improved genomic prediction accuracy. Instead, progress in areas such as representation learning, training population design, and the integration of multienvironment information is likely to have a greater impact on predictive performance than architectural innovation alone.

### Training population composition and relatedness remain dominant drivers of accuracy

Across all models, predictive performance was strongly influenced by the structure of the training population. This observation is consistent with previous studies showing that genomic prediction accuracy is primarily determined by the genetic relatedness between training and validation sets rather than by the specific statistical method employed (Rincent et al. 2012; Daetwyler et al. 2013). When phenotypic variation aligns with realized genomic relationships, even simple linear models can achieve high accuracy, whereas no method can overcome a lack of shared genetic signal between populations. The observed association between phylogenetic signal and prediction accuracy in this study provides further support for this principle. Traits whose variation follows population structure are inherently easier to predict, whereas traits decoupled from relatedness pose a fundamental challenge to genomic prediction regardless of model sophistication.

### Implications for breeding applications and long-term genetic gain

From a practical breeding perspective, the near-equivalence in performance observed among several genomic prediction models suggests that considerations related to implementation should play a central role in guiding model choice. In this regard, factors such as computational efficiency, interpretability, and ease of deployment are of particular importance. rrBLUP and closely related linear methods therefore continue to represent attractive options for routine use in breeding programs, owing to their stable performance, transparency, and well-established statistical foundations. Although ensemble stacking and machine-learning approaches may offer advantages under certain specific conditions, their routine application in breeding pipelines should be supported by clear and consistent evidence of additional benefit. It is also important to recognize that relatively small differences in predictive accuracy among models may not necessarily translate into meaningful differences in realized genetic gain. This is especially relevant in practical breeding scenarios where selection decisions are commonly based on truncation thresholds rather than on continuous optimization of breeding values (Villar-Hernández et al. 2018). Furthermore, an exclusive focus on maximizing short-term genomic gain through aggressive selection strategies may inadvertently accelerate the erosion of genetic diversity within breeding populations. This can be mitigated by using strategies such as supersaturated designs, which are specifically aimed at maximizing the capture of genetic variation within breeding populations (Persa et al. 2023; Virdi et al. 2023). This consideration highlights the importance of integrating genomic prediction approaches with breeding strategies that explicitly account for the management of genetic variance and the maintenance of long-term sustainability (Wolfe et al. 2021; Atanda and Bandillo 2024).

## Conclusion

The present study demonstrates that the accuracy of genomic prediction in mango is governed primarily by the evolutionary structure of the traits rather than by the choice of prediction model. In this context, linear, Bayesian, kernel-based, and ridge-stacked ensemble models exhibited comparable performance across traits, suggesting that more complex algorithms do not necessarily lead to substantial improvements in prediction accuracy. Traits possessing stronger phylogenetic structure, such as FW and blush color, consistently showed higher prediction accuracy; however, traits characterized by weaker phylogenetic signal, such as FF, were found to be inherently more difficult to predict. The results further indicate that rrBLUP, BayesB, and RKHS models were effective at capturing most of the useful genomic information. In contrast, machine-learning and graph-based approaches either contributed marginally or reduced prediction performance. In addition, ridge-based stacking achieved prediction accuracy comparable to that of the best-performing individual models. Still, it failed to provide consistent gains, thereby reflecting the similarity in inductive assumptions among effective base learners. Therefore, the findings of this study suggest that future improvement in mango breeding programs is more likely to be achieved by aligning genomic prediction strategies with underlying evolutionary and genetic constraints rather than by adopting increasingly complex modeling approaches.

## Supplementary Material

jkag124_Supplementary_Data

## Data Availability

All data are presented in table and figure form within the manuscript. The codes are available at https://github.com/GaneshAl/Genomic-prediction. Supplemental material available at G3 online.
