# Supplementary material for: Evolutionary structure constrains genomic prediction accuracy more than model complexity in mango (Mangifera indica L.)
Source: G3 (Bethesda). 2026 May 11;16(7):jkag124. doi: 10.1093/g3journal/jkag124 (PMC13334191; doi:10.1093/g3journal/jkag124)
Supplement: jkag124_Supplementary_Data [file jkag124_supplementary_data.zip › Supplemental_Material_G3-2026-406846.docx]

**Supplementary Table 1: Summary of SNP filtering and quality control steps**

| Steps | Genotypes | SNPs |
| --- | --- | --- |
| Raw dataset | 225 | 16758600 |
| After applying Bi-allelic filter | 225 | 15661181 |
| After removing bad individuals | 224 | 15661181 |
| After removing missing SNPs | 224 | 11246107 |
| After MAF filter | 224 | 7917182 |
| After contig. filter | 224 | 7917182 |
| After genotype imputation | 224 | 6615581 |
| After LD pruning | 224 | 360138 |

**Supplementary Table 2: Pairwise statistical comparisons of prediction accuracy across models**

| **Trait** | **Comparison** | **p-value** | **Significance** |
| --- | --- | --- | --- |
| BC | rrBLUP vs BayesB | 0.101 | ns |
| BC | rrBLUP vs GAT | <0.001 | *** |
| BC | rrBLUP vs RF | <0.001 | *** |
| BC | rrBLUP vs RKHS | <0.001 | *** |
| BC | rrBLUP vs SVR | 0.00391 | ** |
| BC | rrBLUP vs Stacking_Ridge | <0.001 | *** |
| FF | rrBLUP vs BayesB | 0.0461 | * |
| FF | rrBLUP vs GAT | <0.001 | *** |
| FF | rrBLUP vs RF | <0.001 | *** |
| FF | rrBLUP vs RKHS | 0.00290 | ** |
| FF | rrBLUP vs SVR | <0.001 | *** |
| FF | rrBLUP vs Stacking_Ridge | 0.149 | ns |
| FW | rrBLUP vs BayesB | <0.001 | *** |
| FW | rrBLUP vs GAT | <0.001 | *** |
| FW | rrBLUP vs RF | <0.001 | *** |
| FW | rrBLUP vs RKHS | 0.128 | ns |
| FW | rrBLUP vs SVR | <0.001 | *** |
| FW | rrBLUP vs Stacking_Ridge | 0.871 | ns |
| TC | rrBLUP vs BayesB | 0.00244 | ** |
| TC | rrBLUP vs GAT | <0.001 | *** |
| TC | rrBLUP vs RF | <0.001 | *** |
| TC | rrBLUP vs RKHS | 0.0166 | * |
| TC | rrBLUP vs SVR | <0.001 | *** |
| TC | rrBLUP vs Stacking_Ridge | 0.323 | ns |
| TSS | rrBLUP vs BayesB | 0.130 | ns |
| TSS | rrBLUP vs GAT | <0.001 | *** |
| TSS | rrBLUP vs RF | <0.001 | *** |
| TSS | rrBLUP vs RKHS | <0.001 | *** |
| TSS | rrBLUP vs SVR | <0.001 | *** |
| TSS | rrBLUP vs Stacking_Ridge | <0.001 | *** |

ns: not significant (p ≥ 0.05); * p < 0.05; * p < 0.01; *** p < 0.001*
