# Supplementary figures and images for: Evolutionary structure constrains genomic prediction accuracy more than model complexity in mango (Mangifera indica L.)
Source: G3 (Bethesda). 2026 May 11;16(7):jkag124. doi: 10.1093/g3journal/jkag124 (PMC13334191; doi:10.1093/g3journal/jkag124)

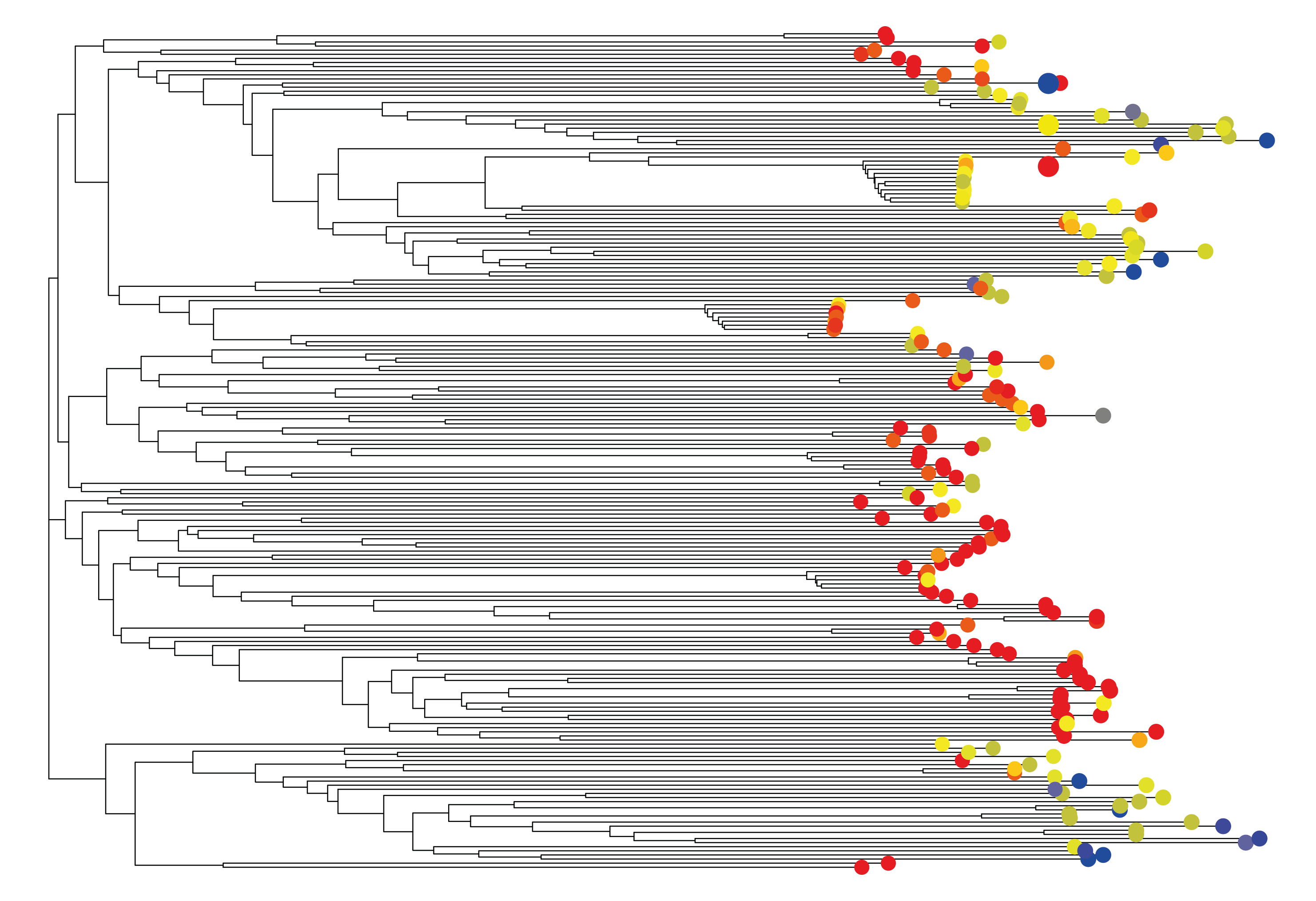

Supplement: jkag124_Supplementary_Data [file jkag124_supplementary_data.zip › Supplemental_Figure_1_G3-2026-406846.png]

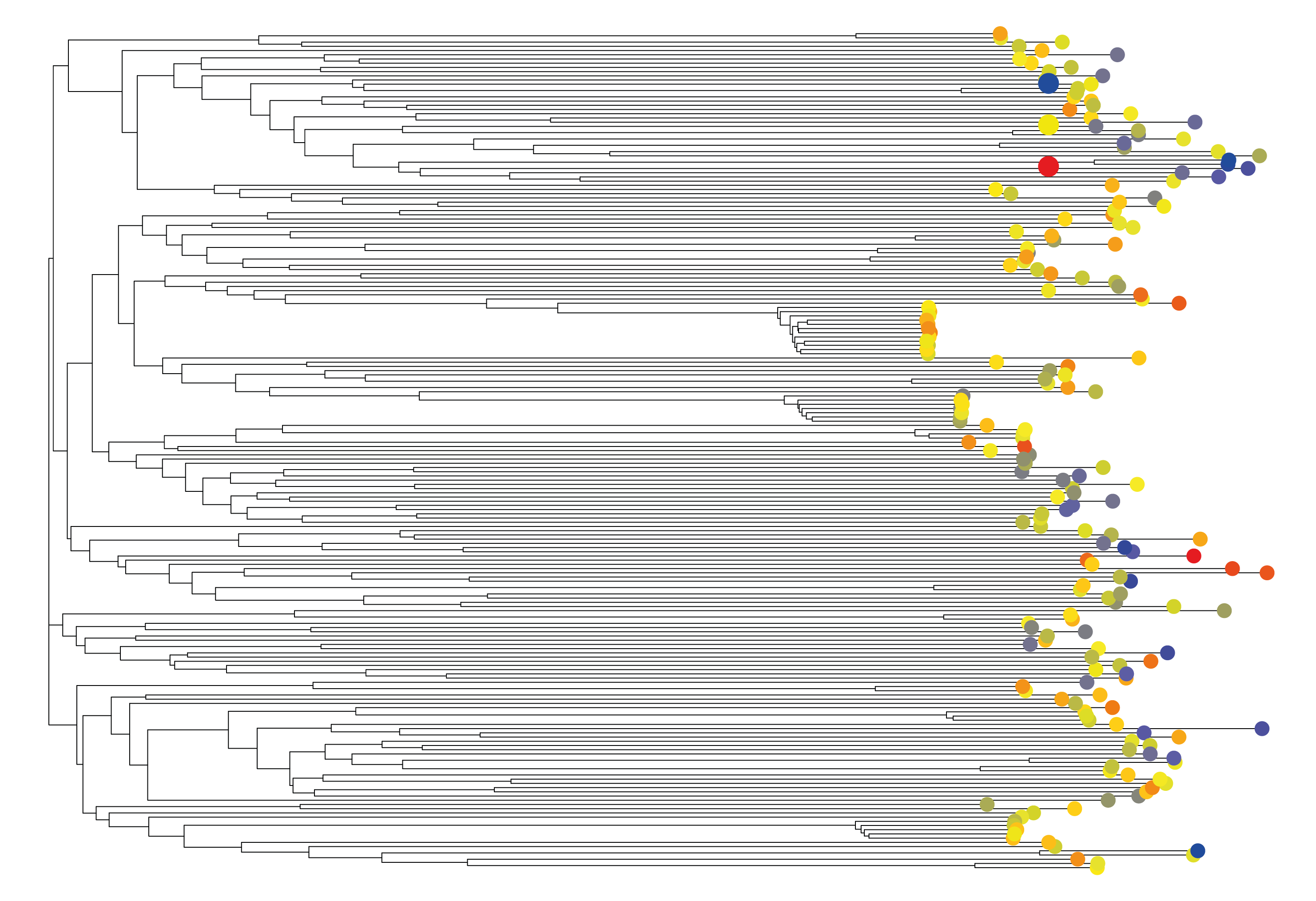

Supplement: jkag124_Supplementary_Data [file jkag124_supplementary_data.zip › Supplemental_Figure_2_G3-2026-406846.png]

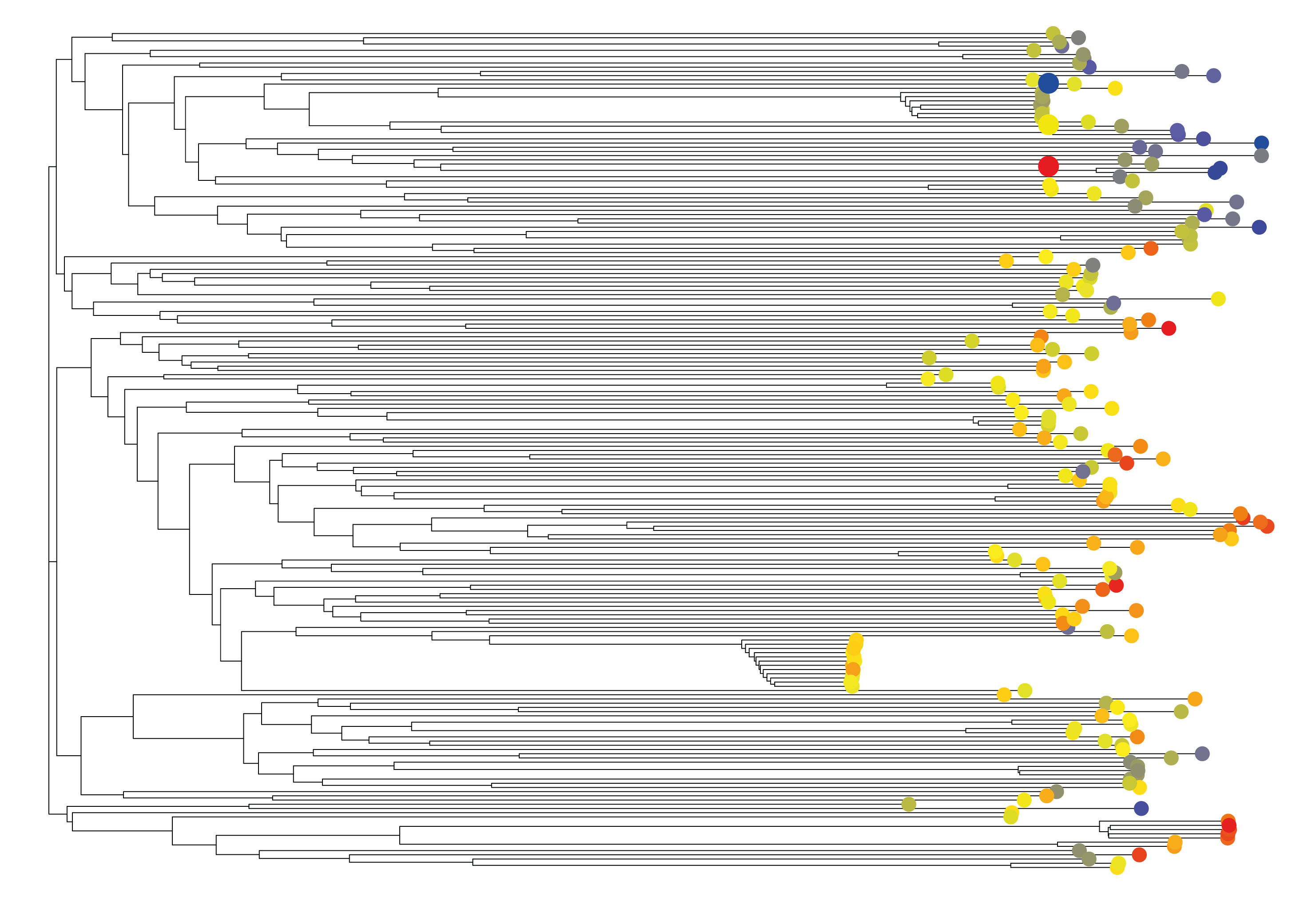

Supplement: jkag124_Supplementary_Data [file jkag124_supplementary_data.zip › Supplemental_Figure_3_G3-2026-406846.png]

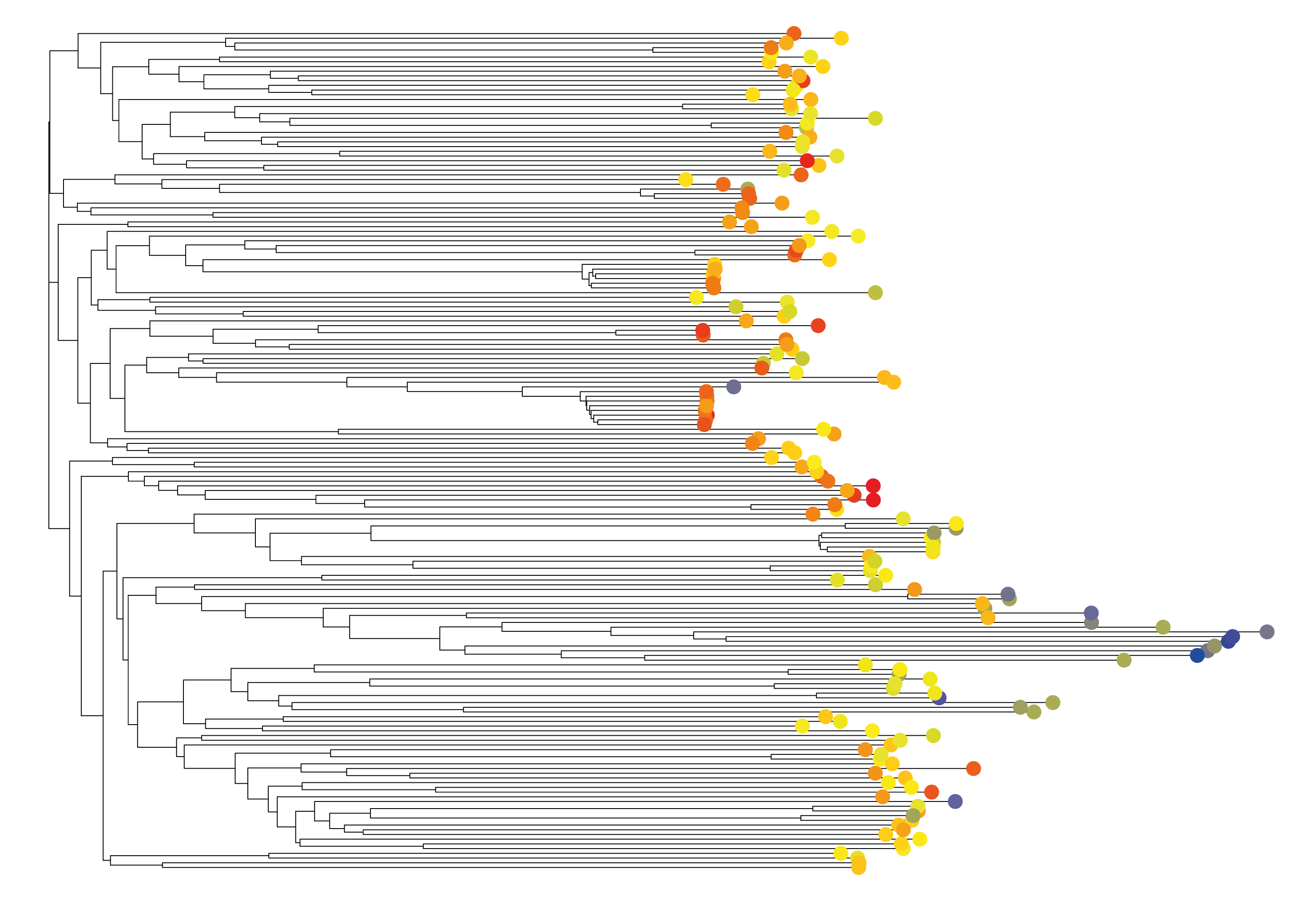

Supplement: jkag124_Supplementary_Data [file jkag124_supplementary_data.zip › Supplemental_Figure_4_G3-2026-406846.png]

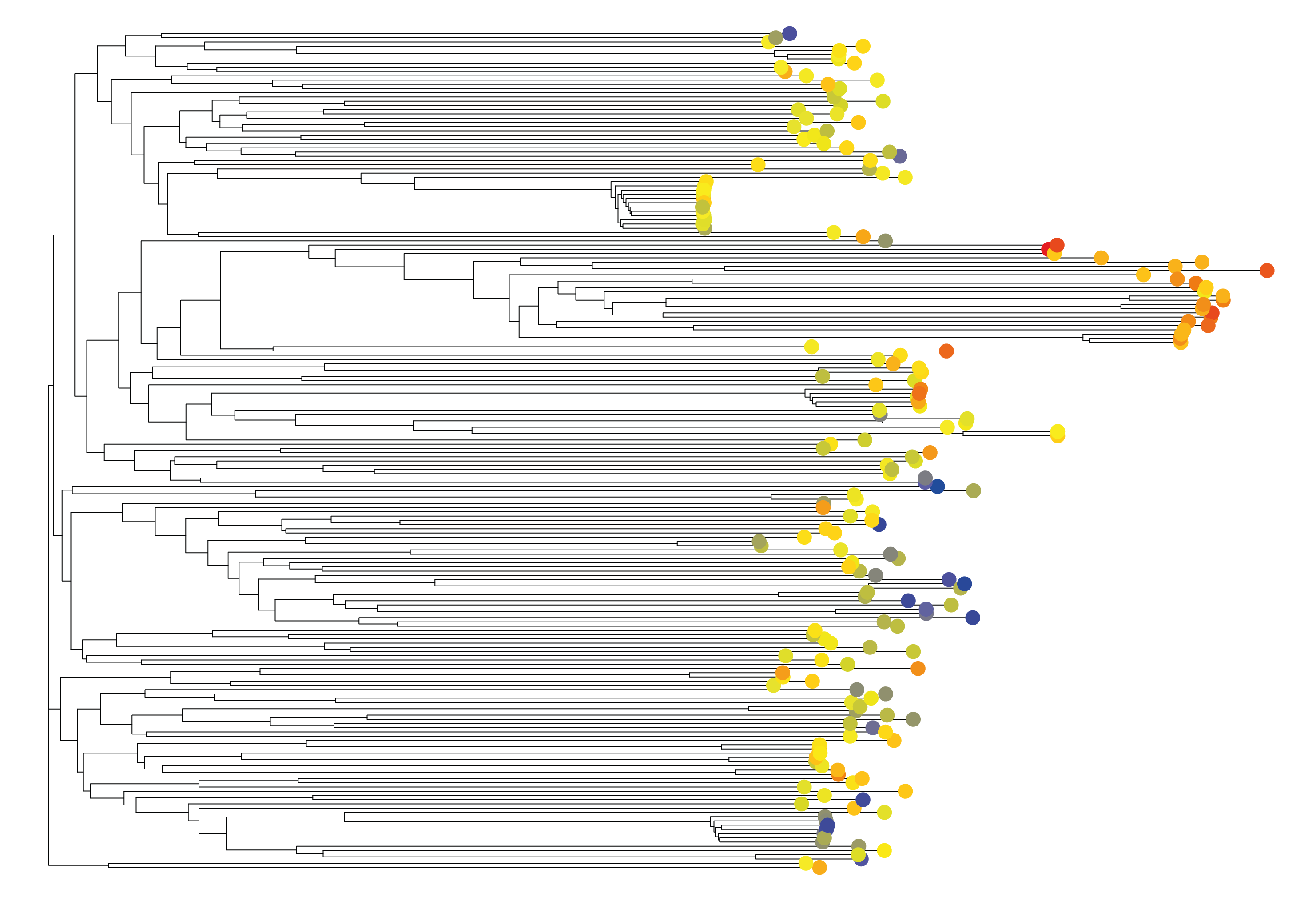

Supplement: jkag124_Supplementary_Data [file jkag124_supplementary_data.zip › Supplemental_Figure_5_G3-2026-406846.png]
